# Durability of Lubricated Icephobic Coatings under Various Environmental Stresses

**DOI:** 10.3390/polym14020303

**Published:** 2022-01-12

**Authors:** Valentina Donadei, Heli Koivuluoto, Essi Sarlin, Petri Vuoristo

**Affiliations:** 1 Materials Science and Environmental Engineering, Faculty of Engineering and Natural Sciences, Tampere University, P.O. Box 589, FI-33014 Tampere, Finland; heli.koivuluoto@tuni.fi (H.K.); essi.sarlin@tuni.fi (E.S.); petri.vuoristo@tuni.fi (P.V.); 2 Tampere Institute for Advanced Study, Tampere University, P.O. Box 1001, FI-33014 Tampere, Finland

**Keywords:** thermal spraying, flame spraying, polymer coatings, lubricated coatings, icephobicity, durability, polymer degradation

## Abstract

Icephobic coatings interest various industries facing icing problems. However, their durability represents a current limitation in real applications. Therefore, understanding the degradation of coatings under various environmental stresses is necessary for further coating development. Here, lubricated icephobic coatings were fabricated using a flame spray method with hybrid feedstock injection. Low-density polyethylene represented the main coating component. Two additives, namely fully hydrogenated cottonseed oil and paraffinic wax, were added to the coating structure to enhance coating icephobicity. Coating properties were characterised, including topography, surface roughness, thermal properties, wettability, and icephobicity. Moreover, their performance was investigated under various environmental stresses, such as repeated icing/deicing cycles, immersion in corrosive media, and exposure to ultraviolet (UV) irradiation. According to the results, all coatings exhibited medium-low ice adhesion, with slightly more stable icephobic behaviour for cottonseed oil-based coatings over the icing/deicing cycles. Surface roughness slightly increased, and wetting performances decreased after the cyclic tests, but chemical changes were not revealed. Moreover, coatings demonstrated good chemical resistance in selected corrosive media, with better performance for paraffin-based coatings. However, a slight decrease in hydrophobicity was detected due to surface structural changes. Finally, paraffin-based coatings showed better resistance under UV irradiation based on carbonyl index and colour change measurements.

## 1. Introduction

In the past decades, passive icephobic solutions, i.e., coatings or surface modifications enabling ice removal, have emerged as potential strategies to face icing problems [1]. Ice accretes and accumulates on surfaces, affecting the operational performances of many engineering applications from aerospace and maritime transportation to renewable energy and power distribution [2,3]. Malfunction, decreased performance, economic losses, and endangerment of human life represent some of the consequences of atmospheric ice accumulating on infrastructure [4]. Current strategies to mitigate icing problems, known as active deicing methods, consist of ice removal from surfaces using external mechanical and thermal loads [5,6,7]. Moreover, ice accretion is prevented or limited by applying chemicals on surfaces, known as anti-icing or deicing fluids. However, these active methods require costly operations, wasting time, energy, and resources while causing environmental pollution [8,9]. Therefore, the current research is oriented towards more sustainable anti-icing strategies.

Icephobic coatings as passive anti-icing strategies are highly desirable as alternative solutions to solve icing problems. However, current durability challenges preclude their applicability in various fields [10,11]. Specifically, icephobic coatings should exhibit permanent icephobic properties under application-related icing conditions. Cyclic icing/deicing tests have been performed to investigate the icing durability of coatings in several studies [12,13,14,15,16]. In addition to icing durability, the stability of coatings has been tested under external loads, such as mechanical [17,18,19,20], chemical [21,22], and thermal stresses and irradiation [10,23,24], to extend their use to real applications [11]. Moreover, recent studies have proposed combining icephobic coatings with active anti-icing methods to mitigate icing problems in harsher icing conditions [4,25,26]. Therefore, more durable passive anti-icing methods are strongly desired, and further research is needed to improve the durability of current icephobic solutions.

The literature presents extensive work on investigating the durability of passive icephobic surfaces, specifically for designs such as superhydrophobic surfaces (SHSs) and slippery liquid-infused porous surfaces (SLIPSs) [11,14,20,22,27,28,29,30,31,32]. However, little attention has been given to assessing the durability of icephobic polymer coatings and composite polymer coatings [10,13,18,33,34]. Therefore, the present study investigates the durability of icephobic polymer coatings under various environmental stresses. Firstly, the durability of coatings is tested under repeated icing/deicing cycles. Secondly, the coating performance is investigated under environmental loads, such as chemical stresses and irradiation, which have not been considered in our previous works [35,36,37]. Coatings are fabricated using a one-step method, previously termed a flame spray process with hybrid feedstock injection [35]. This versatile method was used to produce composite coatings named lubricated icephobic coatings (LICs) [35]. LICs consist of two polymeric components, namely matrix material and lubricating additive. In this study, the matrix material is low-density polyethylene. Two different materials are employed as lubricating additives of the coatings, namely fully hydrogenated cottonseed oil and paraffinic wax. Waxes are generally used in different coating applications to achieve specific surface-related properties, such as glossy or matte appearance and slipperiness [38]. Moreover, they can function as anti-blocking, anti-settling, and anti-sagging agents [38]. More importantly, waxes and polymers, particularly polyolefins, have demonstrated low surface free energy properties [39,40], which can promote reduced ice adhesion strength on smooth polymeric surfaces [41]. Therefore, in this study, waxes are added to the coating structure, aiming at enhancing the icephobic properties of LIC surfaces. 

In summary, this study focuses on (1) producing LICs based on low surface free energy additives by one-step fabrication method, (2) characterising the properties of feedstock materials and obtained coatings, (3) testing the icephobic behaviour of coatings, and (4) assessing the durability of coatings under various environmental stresses, such as repeated icing/deicing cycles, immersion in corrosive environments and exposure to ultraviolet (UV) irradiation. Some significant results indicate that cottonseed oil-based coatings show a better icephobic behaviour than paraffinic wax-based coatings. However, the latter better tolerate corrosive conditions and exposure to UV irradiation.

## 2. Materials and Methods

### 2.1. Materials and Coating Fabrication

Composite polymer coatings were fabricated from commercially available feedstock powders. The primary component of coatings was low-density polyethylene (LDPE) powder (Plascoat LDPE, Plascoat Europe BV, Zuidland, Netherlands). Two different additive powders were selected as the lubricating component of the coatings. The first additive was made of fully hydrogenated cottonseed oil (Lubritab^®^ capsules, JRS PHARMA GmbH & Co. KG, Rosenberg, Germany) and hereafter indicated with the letter C in the sample codes. The second additive was a non-functionalised Fischer–Tropsch hard paraffinic wax (VESTOWAX^®^ H 2050 MG, Evonik Industries AG, Essen, Germany) and hereafter indicated with the letter P.

**Figure 1:**
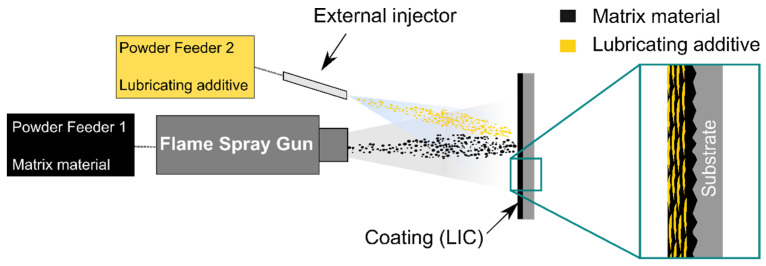
Schematisation of the flame spray process with hybrid feedstock injection to produce lubricated icephobic coatings (LICs).

LICs were manufactured using a flame spray method with hybrid feedstock injection [35], schematically represented in Figure 1. In this method, LDPE was sprayed by an oxygen-acetylene flame spray gun (CastoDyn DS 8000, Castolin Eutectic, Dällikon, Switzerland). This powder was fed using a first powder feeder (Sulzer Metco 4MP, Oerlikon Metco, Wohlen, Switzerland) with nitrogen as a carrier gas. The lubricating additive was sprayed from an external injector mounted on the left side of the gun. The additive was fed using a second powder feeder (PT-10 Twin powder feeder, Oerlikon Metco, Wohlen, Switzerland) with argon as a carrier gas. The spray gun was mounted on a single-arm robot (ABB IRB 4400/60, ABB Robotics, Västerås, Sweden), and the spraying process was automated.

Coatings were deposited on stainless steel substrates (EN 1.4301/2K (4N)), cut in dimensions of 30 mm × 60 mm × 1.5 mm. Substrates were grit-blasted before spraying using aluminium oxide grits (grit size of 54 Mesh). Grit blasting ensures the adhesion of polymer coatings on metal surfaces via a mechanical interlocking mechanism. Substrates were pre-heated by flame, and spraying started when the substrate temperature exceeded the melting range of the LDPE powder at approximately 120 °C. The substrate temperature was monitored using a thermal imaging camera (Ti300 Infrared Camera IR Fusion Technology, Fluke Corporation, Everett, WA, USA). Firstly, the matrix material was sprayed to produce an LDPE coating layer. After the first layer, matrix and additive materials were sprayed simultaneously to fabricate LICs. Coatings with a thickness of approximately 700 µm were deposited on the substrate. Once the coating deposition was completed, some coatings underwent a post-heating treatment by flame. Table 1 provides the process parameters of the flame spray process with hybrid feedstock injection. Table 2 presents the coatings produced in this study with details on employed materials and process parameters. The asterisk in the sample name indicates the post-heating treatment by flame.

Plain additive coatings were fabricated using the external injector of the hybrid feedstock injection system. Deposition of additive coatings was challenging because the melted materials dripped down from the metal substrates during spraying. However, these coatings were produced to better understand the icephobic properties of additives and the icephobic behaviour of corresponding composite coatings.

### 2.2. Characterisation Methods

#### 2.2.1. Feedstock Material Characterization

The morphology of powders was imaged using a scanning electron microscope (SEM, IT-500, JEOL Ltd., Tokyo, Japan). Before the analysis, the powders were sputtered with a thin gold layer to enhance surface conductivity. A secondary electron detector was used to image the powder morphologies. 

Particle size distribution was analysed using a laser diffraction analysis with a dry powder method (LS 13 320 Laser Diffraction Particle Size Analyser, Beckman Coulter, Inc., Brea, CA, USA). The particle size distributions were reported using the annotation −d90 + d10, where d90 and d10 represent the particle sizes below which 90% and 10% of total particles, respectively, are counted.

The chemical characterisation of the feedstock materials was performed using a Fourier-transform infrared spectrometer (Spectrum One FT-IR Spectrometer, Perkin Elmer Instruments, Waltham, MA, USA). This analysis was carried out using an attenuated total reflectance (ATR) sample holder (Universal ATR Sampling accessory, Perkin Elmer Instruments, Waltham, MA, USA) with a diamond crystal. The FTIR spectra were acquired in the wavenumber range 4000 cm^−1^ to 600 cm^−1^ by recording 32 scans with a 4 cm^−1^ resolution. The measurements were repeated three times.

The thermal characterisation was performed using differential scanning calorimetry (DSC, Netzsch DSC214 Polyma, Netzsch, Selb, Germany). Specimens (approx. 10 mg) were placed in a concavus aluminium pan. Dynamic heating was carried out at 20 °C/min from −30 °C to 150 °C in nitrogen atmosphere (40 mL/min nitrogen flow), and glass transition, melting transition, and melting peaks were identified. The thermal stability was investigated by thermogravimetric (TG) analysis (Netzsch TGA209F Tarsus, Netzsch, Selb, Germany). Specimens (approx. 10 mg) were placed in open alumina pans, and dynamic heating was performed at 20 °C/min from 25 to 600 °C in nitrogen atmosphere (20 mL/min nitrogen flow). The thermal stability of the material was estimated by measuring the onset decomposition temperature, T_onset_, of the dynamic TG curve, according to the standard ISO 11358-1. Furthermore, the maximum degradation rate temperature of the powders was estimated at the peak of the first derivative of the TG curve (DTG curve). The results of the thermal analyses were obtained as the average and standard deviation of three measurements.

The thermal oxidation resistance of the feedstock powders was evaluated by measuring the oxidation induction time (OIT) by DSC. This analysis is relevant to predict possible degradation of the feedstock materials in flame spray processes. Specimens (approx. 10 mg) were placed in an open concavus aluminium pan. Firstly, the specimens were heated from room temperature to the oxidation temperature (150 °C or 200 °C) in nitrogen atmosphere at 20 °C/min. After stabilisation of 2 min at the oxidation temperature, the atmosphere of the furnace was changed to oxygen (40 mL/min oxygen flow) for 30 min. The OIT was measured from the gas change until the observed exothermic reaction. The OIT results were obtained as the average and standard deviation of three measurements.

#### 2.2.2. Coating Characterisation

The surface morphology was imaged using a stereomicroscope (MZ7.5, Leica, Wetzlar, Germany) and a scanning electron microscope (SEM, IT-500, JEOL Ltd., Tokyo, Japan). Before the microscopic analysis, the surface samples were sputtered with a thin platinum/palladium layer to enhance surface conductivity. A secondary electron detector was used to image the surface morphologies.

The surface topography was analysed using an optical profilometer (contactless measuring instrument, Alicona Infinite Focus G5, Alicona Imaging GmbH, Raaba, Austria). Roughness parameters (Ra, Sa, Sz) were measured using a 20× objective magnification on 2 mm × 2 mm areas, according to standards ISO 4288 [42] and ISO 25178–3 [43]. Roughness values were obtained by the average and standard deviation of three measurements at different coating locations. 

The chemical characterisation of the coating surfaces was conducted using the same methodology employed for the powders. Surfaces were directly placed on the crystal for the analysis. The measurements were performed in three different locations of the sample surfaces.

The thermal characterization of the coatings was carried out using similar methods to those employed for the thermal analysis of feedstock powders to qualitatively investigate the presence of the additives within the coating structure after coating production. The lubricating additive percentage was qualitatively estimated as the ratio between the enthalpy of fusion of the additive and the total enthalpy of fusion obtained from the DSC curve of the coating.

The wetting behaviour was measured by a droplet shape analyser (DSA100, Krüss, Hamburg, Germany) in controlled conditions (22 °C ± 1 °C temperature and 60% ± 3% relative humidity). Static contact angles were investigated using the sessile drop method by placing 10 μL droplets of ultra-high-purity water (MilliQ, Millipore Corporation, Burlington, MA, USA) onto the surfaces. The apparent water contact angle (WCA) was measured using the tangent method (polynomial fit of droplet shape). Tilting experiments were performed to evaluate the dynamic wetting behaviour of coatings. A 10 μL water droplet was placed onto the coating surfaces, which were tilted until the droplet rolled off, and the tilting angle was recorded. Both WCA and roll-off angle were calculated as the average and standard deviation of five measurements on different sample locations.

Surface free energy (SFE) of coatings was calculated according to the Owens–Wendt–Rabel–Kaelble (WORK) model [44,45,46,47], measuring the static contact angle of water, diiodomethane, and ethylene glycol. This model obtains the SFE of solids as the sum of a dispersive and a polar component. Table 3 presents the polar and dispersive components of the reference liquids used for calculation. The measurements were carried out by depositing ten drops of 3 µL of each liquid on the sample surface. Additionally, surface free energies were measured for the feedstock materials. Feedstock powders were melted and moulded in cylindrical silicon stamps using an oven (drying oven with natural convection, DL 53, DRY-Line^®^, VWR, Darmstadt, Germany). This procedure was carried out to obtain polymer samples in the form of bulks suitable for the SFE measurements.

Icephobic behaviour was measured using the icing facilities in the Ice Laboratory at Tampere University [48]. The test equipment is located in a climate-controlled cold room with monitored temperature and relative humidity (−10 °C ± 1 °C and 80% ± 5%). Mixed-glaze ice was accreted on 30 mm × 30 mm sample areas from supercooled water droplets in an icing wind tunnel (IWiT). After accretion, the ice adhesion strength was measured using a centrifugal ice adhesion test (CAT). In the centrifugal test, specimens with the accreted block of ice are counterweighted and spun with a constant acceleration rate of 300 rpm/s until ice detachment. An acceleration sensor records the value of the rotational speed corresponding to the ice detachment. The shear ice adhesion strength is evaluated as the ratio of the centrifugal force, F [N] at the moment of ice detachment, to the area of the iced surface, A [m^2^]. Equation (1) estimates the shear ice adhesion strength, $\tau_{ice}$ [kPa], as follows:

(1) $$\tau_{ice}=\frac{m_{ice}r\omega^{2}}{A}$$

where $m_{ice}$ [kg] is the known mass of the accreted ice on the specimen, r [m] is the radial spinning length, and ω [rad/s] is the rotational speed. Ice adhesion values were obtained from the average and standard deviation of four parallel samples tested in the icing accretion event. Ice adhesion of a reference surface, namely Teflon tape (TT, 3M™ PTFE Film Tape 5490, 3M, Flemington, NJ, USA), was tested during each ice accretion event to monitor possible ice adhesion variation. The ice adhesion of the reference must necessarily be in the same range of values for a specific ice type to ensure the repeatability and reliability of the icing test and comparison of the results [49].

### 2.3. Durability Tests

#### 2.3.1. Durability under Repeated Icing/Deicing Cycles

The durability of icephobic properties was tested using cyclic icing/deicing tests. Ice was repetitively accreted, and the centrifugal ice adhesion test was performed on the same surfaces four times in total. Four parallel samples of each coating were tested, and the results were calculated as the average and standard deviation of obtained shear ice adhesion values. Variations in ice adhesion were used to assess the durability of the icephobic properties over the cycles. Moreover, changes in surface properties, such as surface morphology, roughness, chemistry, and wettability, were investigated after the cyclic tests. These analyses were conducted to better understand the durability of the icephobic properties.

#### 2.3.2. Durability in Acidic, Saline and Basic Solutions 

The durability of coatings was tested by immersing as-received samples in different chemical solutions. Different aqueous solutions were prepared. An acidic solution was prepared using acetic acid (Acetic acid 99–100% GPR RECTAPUR^®^, VWR Chemicals, Fontenay-sous-Bois, France), and this simulated the interaction of coatings with an acidic rain (pH = 4, considering the average value of the annual rain acidity [50]). A saline solution was prepared using sodium chloride (Sodium chloride, BAKER ANALYZED^®^ ACS, J.T. Baker^®^, VWR Chemicals, Fontenay-sous-Bois, France). The saline solutions mimicked a saline environment with the average sea salt concentration (sodium chloride 35 g/L). A basic solution (pH = 11) was prepared using sodium hydroxide (Sodium Hydroxide, Tamro Oy, Vantaa, Finland), thus recreating the environment of solutions containing cleaning agents, detergents, and ammonia. The pH of the solutions was measured using a pH metre (MU 6100 H, multiparameter instrument, VWR, Darmstadt, Germany). The pH meter was calibrated before each measurement using three buffer solutions with pH of 4, 7, and 10. 

Variations in wetting properties were monitored for all coatings after 7, 15, 30, and 60 days of immersion. The samples were rinsed with de-ionised water, dried with compressed air, and left in controlled conditions (22 °C ± 1 °C temperature and 60% ± 3% relative humidity) for 24 h before the wetting experiments. Possible changes in surface chemistry were investigated by FTIR analysis. TGA analyses were carried out to detect absorbed water in immersed polymers. Surface morphologies were analysed after 60 days of immersion.

#### 2.3.3. Durability under Exposure to Ultraviolet (UV) Irradiation

As-received coatings were artificially aged using simulated solar UV light. The UV irradiation chamber was equipped with four UVA-340 fluorescent lamps. This lamp provides an excellent simulation of natural sunlight in the short-wavelength UV region (ranging from approximately 365 nm to 295 nm). Because the UVA-340 lamp simulates sunlight in the spectrum area that causes the most polymer degradation, this could theoretically ensure better correlation with outdoor test results than other used light sources [38,51]. The dose rate of the chamber was measured as 0.7 W/ m^2^ at the UVB range (290–315 nm), 12.1 W/m^2^ at the UVA range (315–400 nm), and 3.1 W/m^2^ at the visible range (400–600 nm) [52]. The temperature of the UV chamber reached 31 °C when all lamps were functioning. Further technical details on the UV chamber are reported in a previous study [52]. For the UV irradiation tests, samples were exposed for 0, 200, 500, and 1000 h. Changes in chemical composition were investigated using FTIR analysis. The carbonyl index (CI) was used to assess the degree of photodegradation of the coatings. This index was calculated as the ratio between the integrated band absorbances of the carbonyl (C=O) peak from 1850 to 1650 cm^−1^ and the methylene (CH_2_) scissoring peak from 1500 to 1420 cm^−1^, following the specified area under band (SAUB) methodology [53]. Colour changes of the surfaces were observed using visual inspection and a chromameter (CR-200, Konica Minolta Sensing Europe B.V., Nieuwegein, The Netherlands). The chromaticity experiments employed a measuring mode based on L*a*b* coordinates (CIE 1976). In the L*a*b* colour space, L* indicates the colour lightness, a* refers to the red/green coordinate, and b* refers to the yellow/blue coordinate [54]. According to the standard ISO/CIE 11664-4 [55], the colour difference, $\Delta E_{ab}^{*}$, is calculated at 200, 500, and 1000 h with the following Equation (2):

(2) $$\Delta E_{ab}^{*}=\sqrt{{\left(L_{2}^{*}-L_{1}^{*}\;\right)}^{2}+{\left(a_{2}^{*}-a_{1}^{*}\right)}^{2}+{\left(b_{2}^{*}-b_{1}^{*}\right)}^{2}}$$

where $L_{1}^{*},$ $a_{1}^{*}$, and $b_{1}^{*}$ represent the lightness, the red/green coordinate, and the yellow/blue coordinate of the as-received samples, corresponding to 0 h exposure time. $L_{2}^{*},$ $a_{2}^{*}$, and $b_{2}^{*}$ represent the values for the samples after 200 h, 500 h, and 1000 h of exposure time.

## 3. Results and Discussion

### 3.1. Properties of the Feedstock Powders

**Figure 2:**
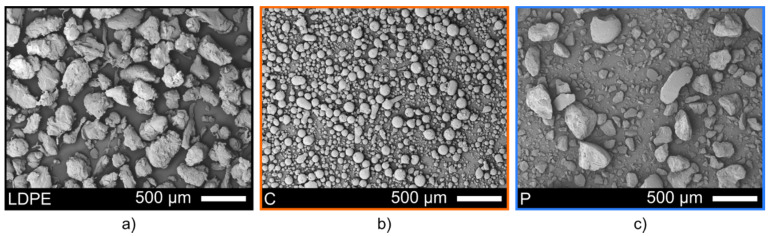
SEM micrographs of the as-received feedstock powders: (**a**) LDPE powder (LDPE), (**b**) fully hy-drogenated cottonseed oil powder (C), and (**c**) hard paraffinic wax powder (P).

Figure 2 shows the morphology of the feedstock powders used to fabricate lubricated icephobic coatings in this study. The LDPE powder (Figure 2a), which constitutes the main component of the coating, was characterised by particle shapes varying from blocky irregular grains to long stretched flakes. The particle analysis revealed a size distribution of −482 + 154 μm. The fully hydrogenated cottonseed oil additive powder (C) showed particles with a spherical shape (Figure 2b) and narrower size distribution of −152 + 34 μm. The hard paraffinic wax (P) presented particles with various irregular shapes (Figure 2c) and a broader size distribution of −450 + 29 µm, as compared to the other additive powder.

**Figure 3:**
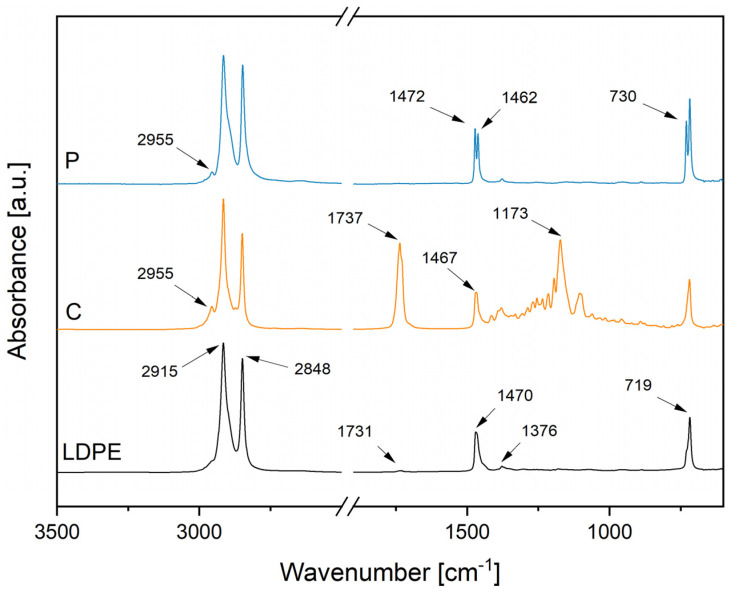
FTIR spectra of feedstock powders with characteristic absorbance peaks. LDPE, C, and P refer to low-density polyethylene, fully hydrogenated cottonseed oil, and paraffinic wax powders, respectively.

Figure 3 presents the FTIR spectra of the feedstock powders. The chemical analysis of feedstock materials aimed at monitoring possible chemical changes occurring in the polymers interacting with the combustion flame during coating fabrication. All the spectra presented typical peaks, characteristics of polymers made of long aliphatic hydrocarbon chains, such as polyethylene [56]. The signals at 2915 and 2848 cm^−^^1^ corresponded to the asymmetric and symmetric stretching of aliphatic hydrocarbons, respectively. The signals at 1472, 1470, 1467, 1462, and 1376 cm^−1^ represented the in-plane vibrations of aliphatic hydrocarbons [56,57]. The signals at 730 and 719 cm^−1^ indicated the rocking of the same vibrational group [56,57]. As expected, the spectra of LDPE and P were remarkably similar due to their identical chemical composition. However, P powder showed double peaks in the range of the in-plane vibrations and rocking of aliphatic hydrocarbons compared to LDPE powder. Similar spectra for paraffinic waxes have been found in previous studies [57,58,59,60]. Additionally, LDPE presented a weak signal at 1731 cm^−1^. This signal probably indicates the presence of conventional stabilisers of undisclosed composition (probably carbonyl products), which are generally incorporated into commercial products [61]. In addition to the aliphatic chain signals, C powder presented two intense absorption peaks at 1737 cm^−1^ and 1173 cm^−1^. These signals are related to the stretching of the carbonyl group (C=O) and the carbon–oxygen bond (C-O) of the ester group, generally present in natural waxes [57,62]. Moreover, the weak signal at 2955 cm^−1^ was present in both additives. This peak arose from the asymmetric stretching of the methyl (CH_3_) in the aliphatic chain [63,64]. However, this small peak could also be associated with =C-H cis stretching in cottonseed oil in case of residual unsaturation (double carbon–carbon bond) after hydrogenation [65].

Table 4 presents the thermal properties of feedstock powders obtained from differential scanning calorimetry and thermogravimetric analyses. The curves are presented in Supplementary Figure S1. 

No glass transitions were revealed for the as-received feedstock powders in the temperature range of the analysis. LDPE and P powders had melting peaks at higher temperatures than C powder. Both additive powders showed two distinct melting peaks. Moreover, in the TG analyses, the higher the T_onset_, the greater the thermal stability of polymers in the considered atmosphere. Therefore, the results indicated improved thermal stability passing from C to P until the LDPE powder. This temperature is essential when considering flame spray processing of polymers, and T_onset_ should not be exceeded to avoid thermal degradation. The maximum degradation temperatures were later utilized to detect the presence of these components in flame-sprayed coatings.

The feedstock materials are fed using inert carrier gasses, such as nitrogen for the matrix materials and argon for the additives. However, the materials are molten by the heat produced from the combustion flame, which generates an oxidative environment. Moreover, when post-heating by flame is performed, the deposited materials are re-melted in contact with the oxidative atmosphere. Considering the complexity of the spraying environment, the resistance of the feedstock powders was further evaluated in oxidative conditions. The OIT results showed no degradation for the feedstock powders exposed to an oxidative atmosphere at 150 °C. However, oxidative degradation was detected at 200 °C almost immediately for LDPE and P, after 1.5 ± 0.2 min and 0.8 ± 0.1 min, respectively. Surprisingly, the degradation of C powder initiated after 27.9 ± 0.3 min. These results indicate a far greater resistance for C than the other feedstock materials. It is well known that the thermal–oxidative resistance of cottonseed oil increases with reduced unsaturation in the chemical structure [66]. Therefore, the fact that our cottonseed oil powder was fully saturated (the commercial product was fully hydrogenated, i.e., all unsaturations were removed) could justify its remarkable oxidative resistance. Oxidation resistance is beneficial for polymers processed using flame spraying, considering the negative influence of thermal degradation on icephobic properties [67]. Moreover, oxidative degradation could be avoided by maintaining the material temperatures below 150 °C during the whole spraying process.

Table 5 reports the surface free energy values measured for the feedstock samples in the form of bulk. Bulk surfaces were relatively smooth, with Ra values ranging from 0.2 to 1.7 µm. Measured SFE values for LDPE and P were similar to the values found in the literature [44,68]. To the best of our knowledge, no SFE has been reported in other studies for fully hydrogenated cottonseed oil. The results showed lower SFE values for the lubricating additives than for the matrix material. In laboratory-scale studies, it has been shown that surfaces with lower SFE demonstrated enhanced icephobic character [41,68,69]. Moreover, similar results were reported in our previous study, where adding the C additive to the LDPE matrix improved the icephobic behaviour of plain LDPE coatings [35].

### 3.2. Composition and Surface Properties of the Coatings

**Figure 4:**
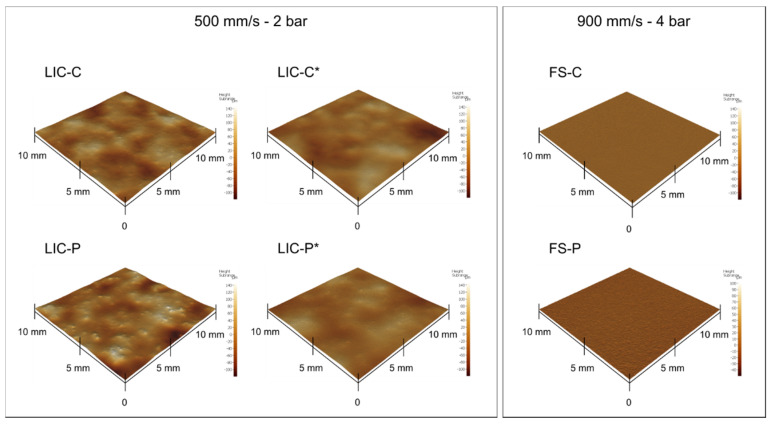
Surface topography of lubricated icephobic coatings (LICs) and flame-sprayed (FS) additive coatings measured using optical profilometry.

Figure 4 presents the surface topography of the coatings together with the employed process parameters. Table 6 provides the areal roughness values (Sa, Sz) for all coating surfaces. From the results, both selected process parameters and thermal properties of feedstock materials influenced the obtained surface topography. If the same process parameters were employed, smoother surfaces were achieved when the lower-melting-temperature additive was sprayed with LDPE (LIC-C compared to LIC-P). Moreover, post-heating by flame smoothened the surfaces by re-melting and re-solidification of polymers, thus producing coatings with a comparable level of roughness (LIC-C* and LIC-P*). Finally, even smoother surfaces were obtained when plain additive coatings were produced, with P-based coatings slightly rougher than C-based coatings (FS-P and FS-C).

**Figure 5:**
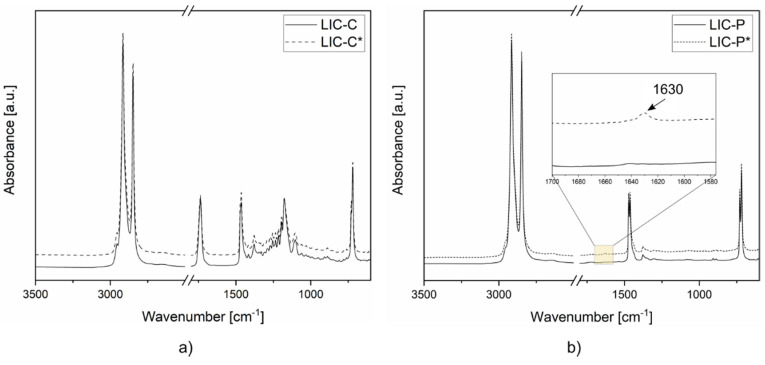
FTIR spectra of lubricated icephobic coatings: (**a**) LIC-C and LIC-C*, and (**b**) LIC-P and LIC-P*. The spectra region is highlighted by a yellow box when chemical modifications are detected.

After coating deposition, chemical characterization of the surfaces was carried out to investigate the presence of the components on surfaces and possible chemical modifications. Figure 5 represents the FTIR spectra of lubricated coatings. The chemical analyses confirmed the presence of matrix material and corresponding additives for all coating surfaces. No significant changes in surface chemistry were revealed for C-based coatings as an effect of the post-heating treatment. Conversely, a small signal at 1630 cm^−1^ was detected for sample LIC-P* compared to LIC-P. This signal might indicate the presence of alkene compounds, which constitutes the initial product of thermal degradation of polyolefins [70]. This behaviour could derive from the lower resistance to thermal oxidation of P compared to C, as demonstrated by previous OIT experiments.

The presence of the additives was further investigated using thermal analyses. Differential scanning calorimetric curves of LIC-C and LIC-C* revealed two distinct melting transitions corresponding to LDPE and C additive. From the ratio between the enthalpies of fusion of additives and coating samples, C content was estimated to be approximately 18 m% ± 1 m% for both coatings. This value approached the theoretical percentage of additive content (20%) determined by the settled feed rates of feedstock powders during spraying. However, the enthalpies of fusion refer solely to the crystalline content of the polymers, and therefore some uncertainty could affect these evaluations. Conversely, LIC-P and LIC-P* coatings showed one melting transition, with melting peaks at intermediate temperatures between the feedstock materials. Identifying P additive was more challenging with this technique, considering the overlapping of melting ranges of P and LDPE. The results obtained from the DSC analyses are provided in Supplementary Figure S2. Similar to DSC results, thermogravimetric curves of LIC-C and LIC-C* evidenced the presence of C additive in the coating structure. The first derivative of the TG curves showed two regions of degradations. The first region was characterized by several shoulders in the degradation range of C. This result evidenced the presence of the minor component C in the coating structure, in accordance with our previous study [35]. The second region referred to the degradation of the main component, LDPE. Conversely, the presence of P additive was hardly identifiable with this technique. One degradation peak was evidenced for LIC-P and LIC-P*. This was due to the overlapping of degradation phenomena of related feedstock materials. The TG and DTG curves of coatings and corresponding feedstock materials are reported in Supplementary Figure S3.

The analysis of wetting properties evaluates the tendency of surfaces to repel water. Wettability has been extensively investigated and used as a screening tool for potential icephobic surfaces [41,71,72,73,74]. Table 7 presents the results of the wetting experiments. For plain flame-sprayed LDPE coatings, water contact angles vary approximately from 84° to 93° for different ranges of roughness [35,67]. From the wetting results of this study, the surface hydrophobicity of plain LDPE coatings was enhanced by the addition of additives. LICs showed intermediate wetting properties, more similar to the additives, with C-based coatings more hydrophobic than P-based coatings. Water contact angles reached approximately 112° and 146° for FS-P and FS-C, respectively, and post-processing by flame had a minor influence on water contact angles. Conversely, the smoothening of LIC-C surfaces lowered the measured roll-off angles, thus improving the water mobility behaviour of post-treated coatings. No roll-off angle was recorded for P-based coatings.

The surface free energy was investigated for LIC-C* and LIC-P* post-heated coatings. SFE values of 22.5 ± 1.9 mN/m and 29.8 ± 0.03 mN/m were measured for LIC-C* and LIC-P* coatings, respectively. SFEs of coatings were intermediate to the corresponding SFEs of feedstock materials (Table 5), with lower values for C-based coatings than P-based coatings. Particularly, SFE of LIC-C* tended towards the SFE value of C additive material, while SFE of LIC-P* was more similar to LDPE matrix material. These results could indicate that the amount of C additive was higher on the surface of post-heated coatings than the P additive. Moreover, the SFE of LIC-P* could tend to LDPE due to possible partial removal of the paraffinic wax from the coating surfaces after post-heating, considering the poorer thermal–oxidative resistance of P compared to C additive, as previously demonstrated by OIT results.

### 3.3. Icephobic Behaviour of Coatings

**Figure 6:**
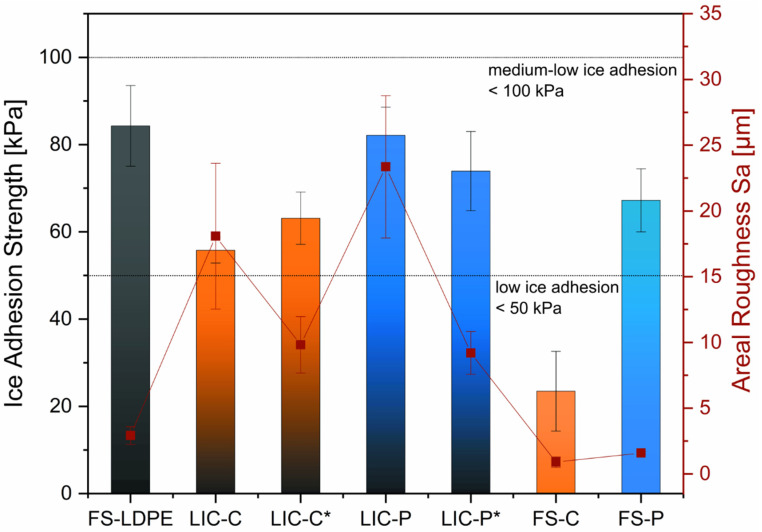
Icephobic behaviour of lubricated icephobic coatings (LICs) and flame-sprayed (FS) feedstock material coatings, measured at −10 °C with mixed glaze ice. Ice adhesion strength is reported on the left axis and corresponding areal roughness Sa on the right axis.

Icephobicity of surfaces is commonly assumed to be the property of repelling ice, delaying ice formation, and/or reducing ice adhesion strength [75]. Moreover, the lower the recorded ice adhesion strength, the higher the icephobicity. In this work, the ice adhesion strength was measured to assess the icephobic behaviour of coatings. For this specific ice adhesion test and these specific icing conditions [14,76], ice adhesion values of 50 kPa and 100 kPa indicate the low and the medium-low ice adhesion limit, respectively. Figure 6 presents the ice adhesion strength of LICs and plain feedstock material coatings with corresponding areal roughness values.

Feedstock material coatings demonstrated medium-low ice adhesion behaviour. Plain LDPE coatings, namely FS-LDPE, showed ice adhesion strength of 84 ± 9 kPa. Plain additive coatings, namely FS-C and FS-P, resulted in ice adhesion values of 23 ± 9 kPa and 67 ± 7 kPa, respectively. Although they showed comparable coating roughness, the ice adhesion values of feedstock material coatings significantly varied, increasing from C to P until LDPE plain coatings. SFE values of corresponding bulk materials similarly rose, passing from C to P until LDPE (Table 5). These results might support the findings of some studies, which evidenced a correlation between low SFE and reduced ice adhesion for smooth polymer coatings [41,68,69]. However, further investigations are needed to establish correlations between ice adhesion strength and SFE values for these materials.

LICs exhibited ice adhesion values between the corresponding plain feedstock material coatings, with C-based coatings slightly more icephobic than P-based coatings. Particularly for C-based coatings, LIC-C and LIC-C* showed medium-low ice adhesion behaviour with a 20% theoretical additive percentage in the coating structure. However, C-based coatings have demonstrated even lower ice adhesion values when a higher additive percentage (30%) has been present in the coating structure [35]. Therefore, the lower additive content could have determined the increased ice adhesion of C-based coatings in this study. Moreover, employed process parameters influence the icephobicity of flame-sprayed polymer coatings [35,67], and this factor needs to be considered when discussing the icephobic behaviour of C-based coatings in this study. For further details on the effect of process parameters on the coating icephobicity, the authors refer to their previous studies [35,67].

Considering the effect of the post-heating treatment by flame, the ice adhesion strength rose approximately 13% for LIC-C* compared to LIC-C. Conversely, the ice adhesion decreased about 10% for LIC-P* compared to LIC-P. However, these results varied within the standard deviation, and the effect of post-heating on the ice adhesion strength was not significant for these materials. This was also confirmed by a *t*-test.

Other factors have been considered to influence the ice adhesion strength, such as surface chemistry, surface roughness, Young’s modulus, shear modulus, and hardness[74,77,78,79], as well as the icing conditions and testing methods [80,81]. Considering surface roughness, various studies have shown that ice adhesion strength rises with surface roughness when surfaces with similar chemistry are considered [82,83]. In this study, post-heating decreased the surface roughness for both coatings with similar surface chemistry, as shown in Figure 6. However, the decrease in roughness seemed to have no significant influence on ice adhesion strength. Specifically, the adhesion values varied within the standard deviation of the test, and a relationship could not be established.

Considering surface wettability, previous studies have demonstrated relationships between apparent water contact angle and ice adhesion strength [74,84]. In contrast, others have claimed no clear correlations between these properties [72,85]. The results showed that C-based coatings were more hydrophobic than P-based coatings, and water droplet mobility was evidenced only for C-based coatings. The latter demonstrated slightly improved icephobicity, thus indicating a possible correlation between wetting properties and icephobic performance [15,74,84]. Additionally, C-based coatings showed lower surface free energy values than P-based coatings, which could justify their enhanced icephobic character [41,68,69]. However, other factors could affect the ice adhesion of these surfaces [35,67,81]. Therefore, further investigations are needed to establish possible correlations between wetting properties, SFE, and ice adhesion strength for these coatings.

### 3.4. Coating Performance under Cyclic Icing/Deicing

In order to test icing performance and durability, researchers have carried out tests based on consecutive icing/deicing actions [12,13,14,15,16,30]. Icing of surfaces is performed using different methods. For example, in one method, water is poured in a mould placed on the surface and then frozen in a freezer at specific temperatures [13,73]. In another method, ice is accreted from supercooled microdroplets hitting the coating surface in the icing wind tunnel under specific icing conditions [16,48,86], representing the icing method employed in this study. Deicing can be pursued through heating [87,88], deformation of the substrate [89], or, more aggressively, applying mechanical loads to the ice itself [14,15,90]. Here, deicing was performed by applying a centrifugal force to the iced samples. Shear ice adhesion strength was recorded at each cycle, and its variations qualitatively indicated the durability of the icephobic properties of coatings. It is essential to underline that the icing durability depends on the methods employed for icing and deicing, the adhesive properties of ice type, and other variables [11]. Moreover, no universal standard has been established yet to test icing durability (icing/deicing procedures, number of icing/deicing cycles, water grade, freezing procedure, ice type, and other test conditions are not defined) [11]. Therefore, discussions on coatings’ icing performance and durability need to be cautiously addressed when comparing results from different studies.

**Figure 7:**
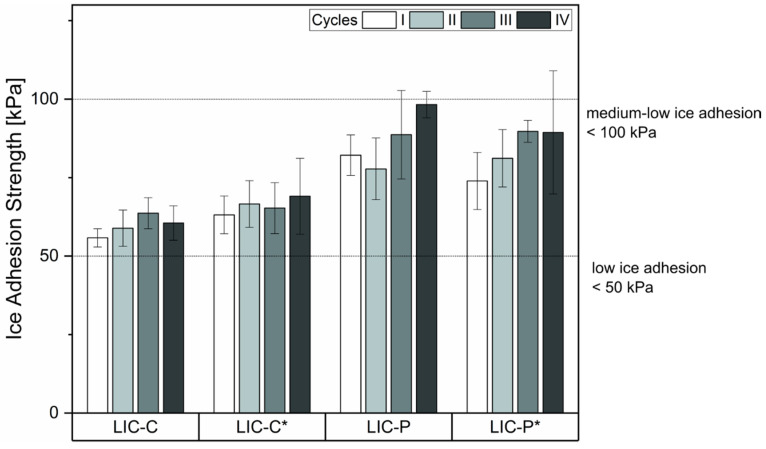
Ice adhesion results for lubricated icephobic coatings (LICs) under four icing/deicing cycles. Ice adhesion strength values at the first cycle were previously reported in Figure 6. C and P refer to fully hydrogenated cottonseed oil and paraffinic wax additives, respectively. The asterisk indicates the post-heating treatment by flame.

Figure 7 presents the shear ice adhesion strength measured at each cycle for LICs. C-based coatings demonstrated slightly lower ice adhesion values and smaller standard deviations than P-based coatings. After four icing/deicing cycles, all coatings retained their icephobicity within the medium-low limit. Particularly for C-based coatings, the four repeated icing/deicing cycles indicated no increasing nor decreasing trends in ice adhesion strength, and the values remained within the standard deviation. This result indicated enhanced icing durability for these coatings compared to our previous studies, where ice adhesion significantly increased over four cycles for C-based coatings [36,37]. This increase over the cycles was evidenced for rougher surfaces containing a higher amount of additive in their coating structure. In the current study, the reduced amount of C additive improved the durability of icephobic properties, maintaining the ice adhesion values within the standard deviation over the cycles.

**Figure 8:**
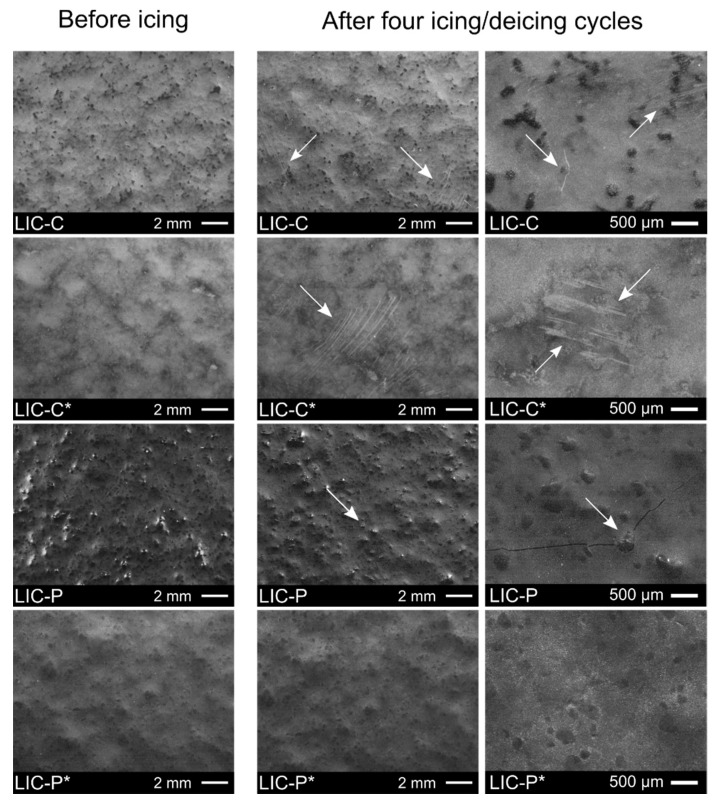
Surface morphologies of lubricated icephobic coatings (LICs) before and after four icing/deicing cycles at different magnification. C and P refer to fully hydrogenated cottonseed oil and paraffinic wax additives, respectively. The asterisk indicates the post-heating treatment by flame. White arrows indicate the presence of surface defects produced during the cyclic tests.

Ice adhesion strength values might vary over the cycles due to changes in surface morphologies and surface chemistry produced during icing/deicing cycles. Figure 8 shows the surface morphologies of all coatings before and after four icing/deicing cycles. The micrographs showed that scratches were visible on C-based coating surfaces, while no evident wear was produced on the polymeric surfaces of P-based coatings. Scratches were probably caused by cyclic shedding of mixed glaze ice via centrifugal forces during icing/deicing. Moreover, it cannot be excluded that a certain degree of surface damage could be produced during sample manipulation. Surface resistance to deicing improved for C-based coatings compared to our previous study, where the whole surfaces were scratched, and the icephobic behaviour significantly decreased over the cycles [36,37]. However, detected scratches did not strongly affect the icephobic behaviour in this study, which was stable over the cycles. Moreover, LIC-C and LIC-P samples showed minor surface cracks after the cycles. In our previous study, cracks were evidenced for some coatings, and these defects influenced the icephobic behaviour of coatings [37]. Here, the number of cracks was so small that they seemed to not affect the surface behaviour of coatings, which retained their icephobicity over the cycles. For additional discussion on the causes of cracks on LICs after cyclic icing/deicing tests, the authors refer to their previous study [37].

**Figure 9:**
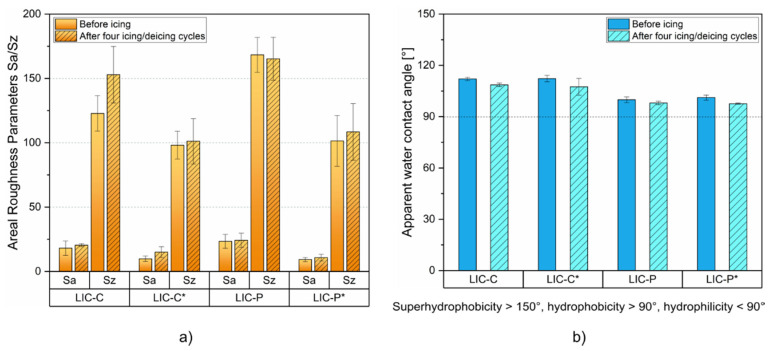
Comparison of surface properties before and after four icing/deicing cycles for LICs: (**a**) areal roughness parameters (Sa and Sz), and (**b**) apparent water contact angles.

Figure 9 compares the variations in surface properties of LICs before and after four icing/deicing cycles. Figure 9a reports Sa and Sz parameters, and Figure 9b reports the apparent water contact angles. From the results on roughness, Sz values of LIC-C samples increased after the cycles. This rise could be justified by the mechanical damages produced on these surfaces, which increased surface roughness [18,37]. However, Sa and Sz mainly varied within the standard deviations for all other coatings. This result indicated that no significant changes in surface roughness were produced for the coatings during the cycles, thus justifying the stable icephobic behaviour of the coatings during the cyclic tests. From the results on wetting properties, only slight decrease in static water contact angle could be revealed for the coatings. This decrease could be related to surface damages produced during the cycles [15]. However, all surfaces retained their hydrophobicity after the cycles, and, in most cases, the variations fell within the standard deviation, similarly to roughness results. In contrast to static contact angle results, water mobility behaviour was affected after the cycles. No roll-off angles were revealed for LIC-C and LIC-C* coatings after the cycles, which initially showed values of approximately 73° and 49°, respectively. This result was due to water droplets pinned into the surface defects formed during the cyclic tests. From the results on surface chemistry, no chemical changes were revealed for the coatings after four icing/deicing cycles, as demonstrated by the FTIR spectra presented in Supplementary Figure S4.

In summary, LICs demonstrated good durability under repeated icing/deicing cycles, retaining their icephobicity below the medium-low ice adhesion limit of 100 kPa. Moreover, employed characterisation techniques revealed no significant changes in surface roughness, water contact angle, and surface chemistry. However, minor surface defects were evidenced for C-based coatings after the cyclic tests, which influenced the water mobility behaviour of surfaces.

The following sections of the manuscript will focus on the durability of LIC-C* and LIC-P* coatings, which showed no mechanical failure (cracks) after the icing/deicing tests. Coating durability in corrosive media and under UV irradiation exposure will be discussed.

### 3.5. Coating Performance in Acidic, Saline and Basic Environments

**Figure 10:**
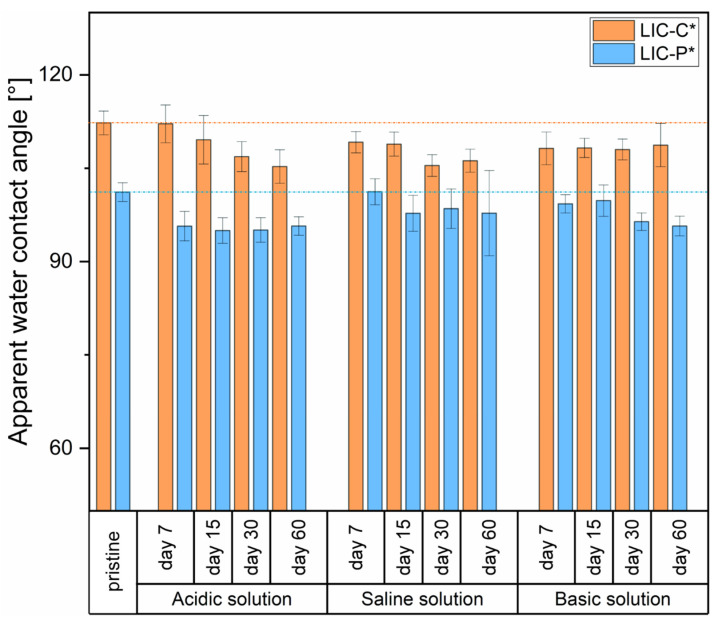
The apparent water contact angle of LIC-C* and LIC-P* samples before and after ageing in acidic, saline, and basic solutions.

Icephobic coatings can contact several environments during their use in outdoor conditions. To simulate these environments and test their durability, three different corrosive media were selected, namely acidic, saline, and basic solutions, and immersion tests were performed. Figure 10 presents the variations of wetting properties after immersion.

Both LIC-C* and LIC-P* samples demonstrated a slight decrease in apparent water contact angles after immersion compared to the pristine ones. This decrease could indicate that variations in surface chemistry and/or surface morphology probably occurred during ageing. In acidic conditions, LIC-C* showed a slight decrease in hydrophobicity until 60 days of immersion. However, water contact angle of LIC-P* samples remained substantially steady from 7 to 60 days after an initial decrease. Conversely, in basic solution, LIC-C* samples demonstrated a better retainment of their wetting properties than LIC-P*, which showed a gradual decrease in hydrophobicity. In saline conditions, both surfaces slightly degraded, and their wetting properties seemed to stabilize after 30 days of immersion. The different behaviour probably derived from the chemical composition of the additives, their chemical resistance in various solutions, surface morphologies of the coatings, and different hydrolytic degradation of each polymer [12]. Besides the slight degradation of wetting properties, all surfaces maintained their hydrophobic character after the immersion tests.

Considering the water mobility properties of LIC-C* surfaces, water roll-off angles increased after 7 days of immersion in all tested conditions. In particular, the roll-off angle rose from 47° ± 5° to 57° ± 6° and 62° ± 3° in saline and basic conditions, respectively. Droplet pinning behaviour was suddenly revealed for the surfaces immersed in acidic solutions after 7 days. No water mobility was detected for LIC-C* surfaces after 15 days of immersion in any of the different conditions. It is well known that water mobility properties are strongly affected by local surface hindrances and heterogeneities, both chemical and morphological, which are encountered by water droplets sliding onto surfaces [91,92,93]. Therefore, the increased roll-off angle value and consequent pinning into the surfaces could indicate the formation of defects on the coating surfaces. Furthermore, repeated cycles of immersion, drying, wetting analyses, and samples manipulation could have produced surface damage, and these actions could influence the wetting results.

**Figure 11:**
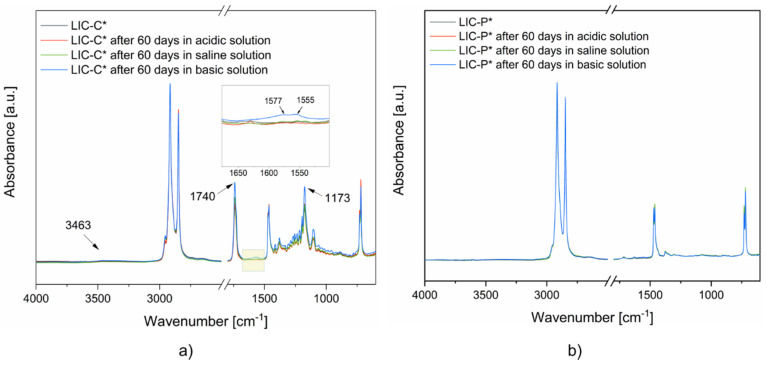
FTIR spectra of LICs surfaces after ageing in different environments: (**a**) LIC-C*, and (**b**) LIC-P*. The regions of the spectra are highlighted by yellow boxes when chemical modifications are detected.

After 60 days of immersion in different conditions, surface chemical analyses were carried out for LIC-C* and LIC-P*. Figure 11 shows the FTIR spectra of the as-received coatings and after 60 days of immersion in different solutions.

The FTIR spectra of LIC-C* coatings (Figure 11a) demonstrated no relevant changes for the samples immersed in acidic and saline environments. However, minor variations in the chemical structure were evidenced for basic solutions experiments. Weak signals were present in the region between 1650 and 1500 cm^−1^. These probably corresponded to the formation of alkene bonds [94,95]. Moreover, increased intensities were noticed for the signals related to the ester bonds at 1740 and 1173 cm^−1^. Furthermore, a weak signal was present in the regions between 3700 and 3200 cm^−1^. These probably corresponded to the formation of hydroxyl bonds [94,95]. Hydroxyl bonds and increased intensity of ester bonds could justify the presence of hydrolysis products of esters, which mainly consist of carboxylic acids [95].

Considering the FTIR spectra of LIC-P* coatings (Figure 11b), no significant chemical changes were revealed for the surfaces in contact with different solutions. Moreover, no absorbed water in the coating structures was detected from thermogravimetric experiments, as shown in Supplementary Figure S5. These results indicated that both surfaces demonstrated significant chemical resistance to water absorption in the tested environments. However, the TG curves related to LIC-C* samples showed some deviations from the pristine samples, which were not visible for the LIC-P* coatings. These deviations could indicate some degradation process for the polymers of LIC-C*, most probably for the additive C. However, further analyses are needed to support these results.

**Figure 12:**
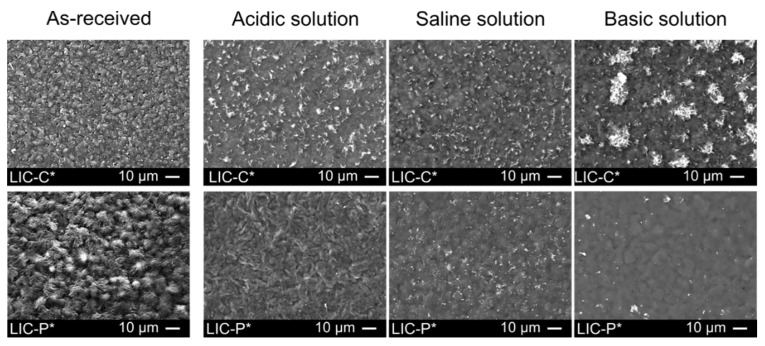
Micrographs of the surface morphologies of LICs after 60 days of immersion in acidic, saline, and basic solutions.

After 60 days of immersion, coating morphologies were further analysed at the microscale level to investigate their durability in the corrosive media. Figure 12 presents the surface morphologies of the coatings after 60 days in acidic, saline, and basic solutions.

As can be seen from the micrographs, the surface morphologies of as-received coatings varied after immersion. Small protuberances characterised as-received surfaces. No similar features were revealed on the coating surfaces after immersion, and structural changes occurred for the surfaces in all solutions. Considering LIC-C* samples, surface morphologies revealed the presence of flake-like structures after immersion. These flakes probably derived from the hydrolytic degradation of C additive, which could be the most sensitive component to hydrolysis. Moreover, larger regions of flake-like structures were noticed for the samples immersed in basic solutions. Immersion tests produced surface morphologies and surface chemistry variations, which generally influence icephobicity [77,96,97]. In our study, the removal of wax from the coating surfaces might cause a decrease in icephobic properties for the aged coatings, as demonstrated for coatings with the lower amount of C additive in our previous studies [35,37]. However, further investigations are necessary to establish these correlations.

Considering LIC-P* samples, structural changes on the surfaces were noticed after immersion. Finer structures were produced after immersion in acidic solutions compared to saline and basic environments. These results could justify the reduced hydrophobicity of the coatings after immersion compared to the as-received ones. However, the wetting behaviour cannot be fully explained using the surface micrographs because these provide no information on the surface chemistry of coatings after immersion.

Degradation of polymers due to water, namely hydrolysis, can lead to cross-linking and porous structures on the exposed polymer surface, thus causing surface structural changes [12]. This phenomenon is enhanced if water-sensitive functional groups are present in the polymers [12,95]. Ester groups, typical of C additive, are more prone to hydrolysis than primary and secondary alkyl groups, which characterize both LDPE and P additive [12,94]. This aspect could justify the small hydroxyl signal evidenced for all aged LIC-C*, which was not revealed for LIC-P* surfaces. However, hydrolysis of esters occurs slowly, even in boiling water. If bases and acids are added to water, the reaction is slightly accelerated but still slow if conducted at room temperature [95]. Therefore, minor chemical degradation was justified considering the current experimental conditions.

Furthermore, other factors could influence hydrolysis, such as hydrophobicity, surface porosity, and mechanical stresses [12]. Concerning hydrophobicity, the tendency for hydrolysis decreases as the hydrophobicity of the surface increases [12]. Therefore, the higher water-repellent properties of LIC-C* samples could have contributed retarding the hydrolysis reaction, despite the presence of ester bonds.

In summary, samples demonstrated good durability in different corrosive environments. LIC-P* demonstrated better resistance in the selected conditions due to its chemical composition, being less affected by hydrolysis reactions. After immersion, structural changes to surfaces were revealed for both samples, which could decrease coating hydrophobicity.

### 3.6. Coating Performance under Exposure to Ultraviolet (UV) Irradiation

Icephobic coatings are primarily used in outdoor conditions, where they are exposed to UV irradiation. Exposure to UV light causes polymers to degrade, thus decreasing their performance and shortening their lifetime [98]. Therefore, understanding the mechanism of polymer degradation under UV exposure is necessary to further improve the durability of icephobic coatings under these conditions.

**Figure 13:**
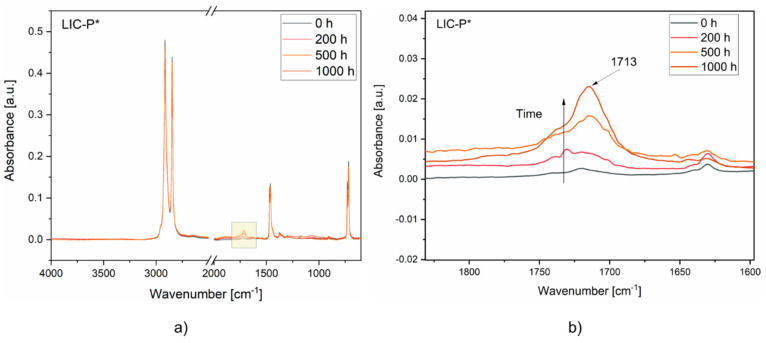
FTIR spectra of the coatings at different exposure times to UV irradiation: (**a**) LIC-P* spectra, (**b**) LIC-P* spectra enlarged in the region of interest. The spectra region is highlighted by a yellow box when chemical modifications are detected.

Figure 13 compares the FTIR spectra of LIC-P* coatings at different times of UV exposure. According to the results, aged coating surfaces showed an increased signal in the region between 1850 and 1650 cm^−1^. The signal gradually rose with the exposure time until 1000 h. This signal was attributed to the formation of carbonyl groups in the polymer structure. In particular, the broad peak with the highest intensity signal at 1713 cm^−1^ indicated the presence of several oxidation products, probably including carboxylic acid, carboxylic ester, and carboxylic anhydride [99]. These results are in accord with previous studies on the photodegradation of polyolefins [99,100,101].

**Figure 14:**
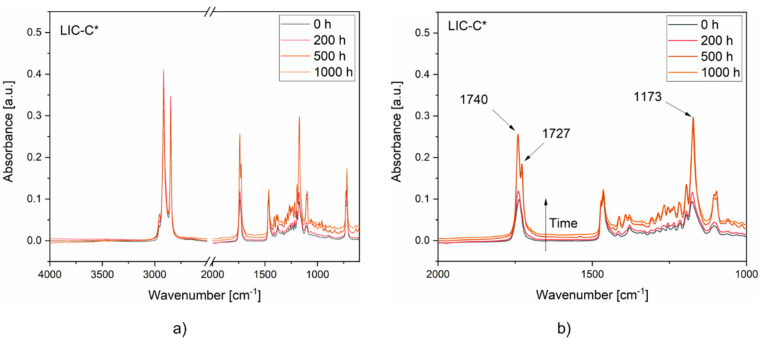
FTIR spectra of the coatings at different exposure times to UV irradiation: (**a**) LIC-C* spectra, (**b**) LIC-C* spectra enlarged in the region of interest.

Figure 14 compares the FTIR spectra of LIC-C* coatings at different exposure times. From these spectra, the signal intensity gradually rose with exposure time. After 200 h, a slight increase was revealed in the peak intensity at 1737 cm^−1^ and 1173 cm^−1^. This rise qualitatively corresponded to the increased stretching signal of the carbonyl bond (C=O) and the carbon–oxygen bond (C-O) of the ester group. After 500 h, the signal intensities of these bonds further increased. In particular, the peak at 1737 cm^−1^ shifted and divided into two peaks at 1740 and 1727 cm^−1^. The first signal at 1740 cm^−1^ corresponded to increased carbonyl bonds, and the second signal at 1727 cm^−1^ represented the formation of carboxylic acids, as reported in a previous study [102]. These results indicated an increased amount of oxidation products due to photo-oxidation, as reported for natural and synthetic waxes in other studies [102,103,104]. Therefore, the photo-oxidation of LIC-C* coatings was mainly related to the degradation of C additive under UV exposure. After 1000 h, no new peaks were detected in the spectra compared to 500 h of exposure.

**Figure 15:**
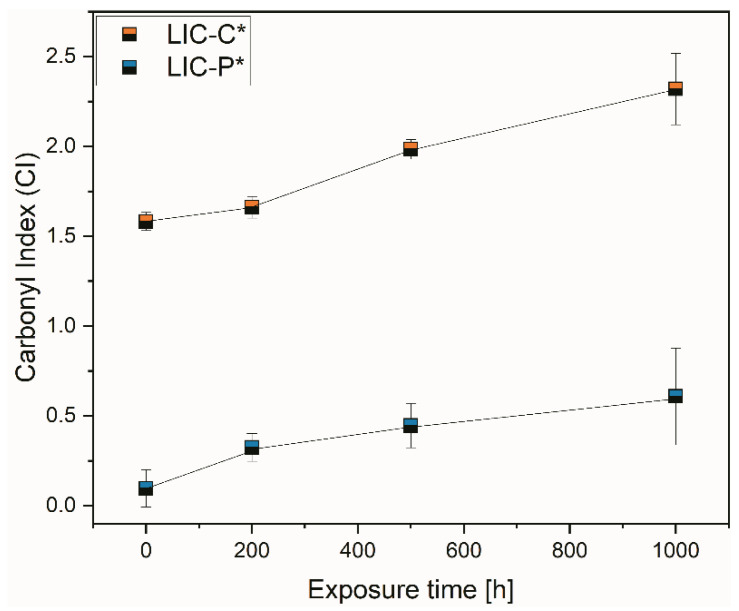
Comparison of carbonyl indices (CI) of LIC-C* and LIC-P* coatings calculated using the specified area under band (SAUB) methodology.

Figure 15 compares the carbonyl indices of LIC-C* and LIC-P* coatings at different exposure times to UV irradiation. The carbonyl index represents one of the most used metrics in polymer chemical degradation studies, considering that carbonyl compounds generally constitute the main product of degradation reactions [52]. The carbonyl index of LIC-C* coatings rose with increasing exposure time. Similarly, the carbonyl index of LIC-P* gradually increased with exposure time, in accordance with FTIR analyses.

**Figure 16:**
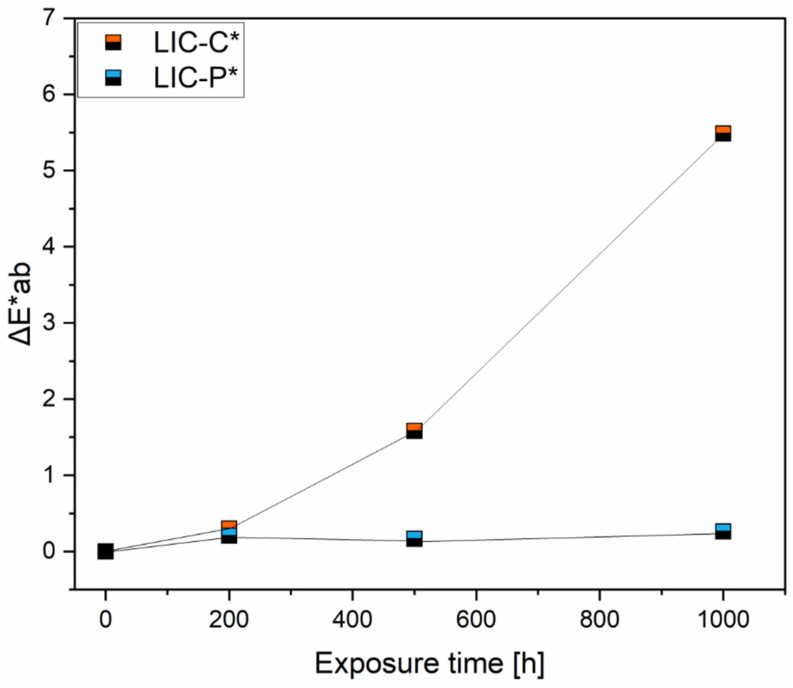
Comparison of colour changes for LIC-C* and LIC-P*coatings at different exposure times to UV irradiation.

Moreover, photo-oxidation under UV irradiation produced colour changes in the coating surfaces. Visual inspection of the coatings detected no significant colour changes of the surfaces, probably because of the black colour of the main component, LDPE. Therefore, a chromameter was used for the analysis. Figure 16 compared the variation of colour, $\Delta E_{ab}^{*}$, for the coatings at different times of exposure.

Colour change value increased with exposure time for LIC-C* coatings compared to LIC-P* coatings, which demonstrated stable behaviour over the time of exposure. Similar results on colour change have been reported for aged polyethylene samples in other research [51]. Colour observations of aged samples correlated with the results of FTIR spectra. The more prominent colour variation was observed when a significant change was revealed in the FTIR spectra for LIC-C* and vice versa for LIC-P*. These results are in accordance with a previous study on weathered polymers and waxes [103].

Considering the sensitivity of chemical bonds to UV irradiation, carbonyl groups of ester bonds generally act as UV light-absorbing groups [94]. Moreover, ester bonds are more vulnerable to UV than primary and secondary alkyl groups typical of polyolefins [12]. As demonstrated by FTIR analyses, ester bonds characterised the chemical structure of fully hydrogenated cottonseed oil. Therefore, the presence of ester bonds could justify the higher tendency to photodegradation of C coatings compared to P coatings, although the presence of absorbent groups is not necessarily decisive for degradation [94,104]. Moreover, non-degraded, pure polyolefins should be photo-chemically resistant, considering that they theoretically do not absorb any UV light. However, UV light can be absorbed if any unsaturation or carbonyl groups are formed during manufacturing or additives are added [94]. Previous results presented in Figure 3 and Figure 5b demonstrated the presence of weak signals at 1731 and 1630 cm^−1^. These corresponded to possible carbonyl products in the feedstock material and alkenes after coating production in LIC-P*, respectively. Their presence could have accelerated the photodegradation reaction in LIC-P* coatings. However, LIC-P* samples better withstood degradation under UV irradiation than LIC-C* samples, and the latter showed better performances in icing conditions.

Surface photo-oxidation was evidenced for both coatings after UV irradiation, with C-based coatings more damaged than P-based coatings. Our previous study demonstrated that ice adhesion increased with surface oxidation for plain LDPE coatings [67]. The higher the oxidation degree of the surfaces, the greater the measured ice adhesion strength of coatings [67]. Moreover, a recent study demonstrated that UV ageing of polyurethane-based coatings negatively influenced their icephobic behaviour [10]. However, further investigations are needed to determine the influence of UV ageing on the icephobicity of these coatings.

## 4. Conclusions

Lubricated icephobic coatings (LICs) were fabricated using the flame spray method with hybrid feedstock injection. This one-step method allowed fast and scalable fabrication of LICs, which were composite polymer coatings made here of the main component, namely low-density polyethylene (LDPE), and two minor lubricating additives, namely fully hydrogenated cottonseed oil (C) and paraffinic wax (P). The choice of lubricants was based on chemical compatibility with the matrix material and surface free energy property. The latter property was considered to enhance the icephobic behaviour of flame-sprayed LDPE coatings. The first part of the study focused on characterising the properties and the icephobic behaviour of coatings. Employed process parameters and materials influenced the surface roughness and wetting behaviour of coatings. Post-heating reduced surface roughness, resulting in improved water droplet mobility. Little thermal degradation was revealed for P-based coatings after post-heating. Concerning icephobic behaviour, all LICs exhibited medium-low ice adhesion values at the first icing/deicing cycle. In general, slightly lower ice adhesion values were revealed for C-based coatings than for P-based coatings, according to the icephobicity of corresponding additive coatings. Moreover, all LICs showed stable icephobic behaviour over the cycles, maintaining their adhesion values below the low-medium limit of 100 kPa. Minor scratches were detected for C-based coatings, which lost their water mobility behaviour after the cycles. However, these surface damages caused no substantial changes in surface roughness, wettability, and icephobicity. Moreover, no chemical changes were produced for all surfaces after the cycles.

The second part of the study investigated the durability of selected coatings in acidic, saline, and basic environments and under exposure to ultraviolet (UV) irradiation. P-based coatings demonstrated excellent chemical resistance in selected corrosive environments, while C-based coatings experienced minor hydrolytic degradation, especially in basic solutions. However, wetting performance slightly decreased for all coatings due to surface structural changes revealed after immersion, but coating hydrophobicity was retained. Moreover, P-based coatings better withstood UV irradiation exposure compared to C-based coatings. The chemical structure of the C additive was characterised by ester bonds, which were more sensitive to UV irradiation. Carbonyl index and colour change measurements confirmed this result.

In summary, C-based coatings demonstrated better icephobicity and stability under repeated icing/deicing cycles compared to P-based coatings. However, the latter showed enhanced resistance to wear against ice shedding, immersion in selected corrosive media, and exposure to UV irradiation. Degradation under the studied environmental stresses mainly resulted in oxidation of the polymeric surfaces. These findings have significant implications in developing icephobic coatings for outdoor applications, thus highlighting the importance of material combinations in the coating structure. Future studies will investigate the effect of ageing in various environments on the icephobicity of coatings to further develop LICs.

## Figures and Tables

**Table 1:** Process parameters of the flame spray process with hybrid feedstock injection.

Process Parameters	Value
CastoDyn DS 8000—flame spray gun	
Nozzle model	SSM10
Combustion gasses	
Oxygen pressure [bar]	4.0
Acetylene pressure [bar]	0.7
Gun spray distance [mm]	250
Step size [mm]	5
Sulzer Metco 4MP-850D dual powder feeder—matrix powder	
Matrix material feed rate [g/min]	26
Carrier gas (nitrogen) flow rate [L/min]	10
Carrier gas (nitrogen) input pressure [bar]	5
PT-10 twin powder feeder—additive powder	
Lubricating additive material feed rate [g/min]	8
Carrier gas (argon) flow rate [L/min]	6

**Table 2:** List of lubricated icephobic coatings (LICs) and flame-sprayed (FS) feedstock material coatings with details on employed materials and process parameters. LDPE, C, and P refer to low-density polyethylene, fully hydrogenated cottonseed oil, and paraffinic wax powders, respectively.

Sample	Materials	Gun Traverse Speed [mm/s]	Gun Air Pressure [bar]	Post-Heating [Y/N]
FS-LDPE	LDPE	500	0	Y
LIC-C	LDPE + C	500	2	N
LIC-C *				Y
LIC-P	LDPE + P			N
LIC-P *				Y
FS-C ^1^	C	900	4	N
FS-P ^1^	P			N

* Coatings were post-heated using the flame of the spray gun; ^1^ Additive coatings (FS-C and FS-P) were fabricated using the external injector of the hybrid feedstock injection system.

**Table 3:** Surface tension components of the reference liquids from the substance database of Krüss Advance software.

Reference Liquid	Chemical Formula	Surface Tension at RT [mN/m]	Dispersive Component [mN/m]	Polar Component [mN/m]
water	H_2_O	72.8	21.8	51
diiodomethane	CH_2_I_2_	50.8	50.8	0
ethylene glycol	C_2_H_6_O_2_	47.7	26.4	21.3

**Table 4:** Thermal properties of low-density polyethylene (LDPE), fully hydrogenated cottonseed oil (C), and paraffinic wax (P): temperature at the melting peaks (T_melting peak_) from the DSC analyses, onset decomposition temperatures (T_onset_) from the TG curves, and maximum degradation temperatures (T_max degradation_) from the DTG curves.

Sample	T_melting peak_ [°C]	T_onset_ [°C]	T_max degradation_ [°C]
LDPE	111.9 ± 0.6	458.2 ± 1.3	481.1 ± 1.9
C	55.6 ± 0.5	402.5 ± 4.6	437.1 ± 1.7
	68.9 ± 2.3		
P	100.3 ± 0.2	435.0 ± 7.0	480.2 ± 1.7
	118.8 ± 0.6		

**Table 5:** Surface free energy (SFE) values with dispersive and polar components of low-density polyeth-ylene (LDPE), fully hydrogenated cottonseed oil (C), and paraffinic wax (P), in the form of bulk.

Feedstock Material	SFE [mN/m]	Dispersive Component [mN/m]	Polar Component [mN/m]
LDPE	30.41 ± 1.25	29.14 ± 0.64	1.27 ± 0.61
C	19.77 ± 1.48	19.15 ± 1.19	0.62 ± 0.28
P	22.84 ± 0.04	22.19 ± 0.03	0.64 ± 0.01

**Table 6:** Average and standard deviation (SD) of the areal roughness parameters evaluated for lubricated icephobic coatings (LICs) and flame-sprayed (FS) additive coatings: average height (Sa) and maximum height (Sz).

Sample	Sa [µm] ± SD [µm]	Sz [μm] ± SD [μm]
LIC-C	18.1 ± 5.5	122.8 ± 13.8
LIC-C*	9.8 ± 2.1	98.1 ± 10.8
LIC-P	23.4 ± 5.4	168.3 ± 13.5
LIC-P*	9.2 ± 1.6	101.5 ± 19.8
FS-C	0.9 ± 0.4	64.2 ± 10.6
FS-P	1.6 ± 0.1	74.1 ± 6.3

**Table 7:** Results of the wetting experiments for lubricated icephobic coatings (LICs) and flame-sprayed (FS) additive coatings.

**Sample**	**Water Contact Angle[°]**	**Water Roll-Off Angle[°]**
LIC-C	112.1 ± 1.0	73.2 ± 3.9
LIC-C*	112.3 ± 1.9	48.6 ± 5.2
LIC-P	99.9 ± 1.7	>90
LIC-P*	101.2 ± 1.5	>90
FS-C	146.3 ± 3.0	31.4 ± 1.4
FS-P	112.6 ± 1.7	>90

## Data Availability

Data from this study are available upon request from the corresponding author.

## Supplementary Materials

The following are available online at www.mdpi.com/2073-4360/14/2/303/s1, Figure S1: Thermal properties of the matrix (LDPE) and lubricating additives (C and P) powders: (a) melting range with peak melting temperatures from the DSC analysis, and (b) onset decomposition temperatures (Tonset) from the TG curves and maximum degradation temperatures from the DTG curves; Figure S2: Curves obtained from the DSC experiments indicating the melting transitions related to (a) coatings LIC-C and LIC-C* and corresponding feedstock powders (LDPE and C), and (b) coatings LIC-P and LIC-P* and corresponding feedstock powders (LDPE and P); Figure S3: Curves obtained from the TG experiments indicating the mass percentage decrease as a function of temperature for (a) coatings LIC-C and LIC-C* and related feedstock powders (LDPE and C) and (c) coatings LIC-P and LIC-P* and related feedstock powders (LDPE and P). The degradation peaks evaluated from the first derivative of TG curves (DTG) are related to (b) LIC-C, LIC-C*, LDPE and C, and (d) LIC-P, LIC-P*, LDPE and P; Figure S4: The FTIR spectra of the coating surface before icing/deicing cycles (solid line) and after four icing/deicing cycles (dash line). The graphs correspond to samples LIC-C (a), LIC-C* (b), LIC-P (c), and LIC-P* (d); Figure S5: Comparison of the thermogravimetric curves of the coatings before and after 60 days of immersion in acidic solution (a,b), in saline solutions (c,d), and basic solutions (e,f).
