# Supplementary material for: Durability of Lubricated Icephobic Coatings under Various Environmental Stresses
Source: Polymers (Basel). 2022 Jan 12;14(2):303. doi: 10.3390/polym14020303 (PMC8779144; doi:10.3390/polym14020303)
Supplement: Supplementary file 1 [file polymers-14-00303-s001.zip › polymers-1534079-supplementary.pdf]

polymers

MDPI

# Supplementary Material: Durability of Lubricated Icephobic Coatings under Various Environmental Stresses

**Valentina Donadei, Heli Koivuluoto, Essi Sarlin and Petri Vuoristo**

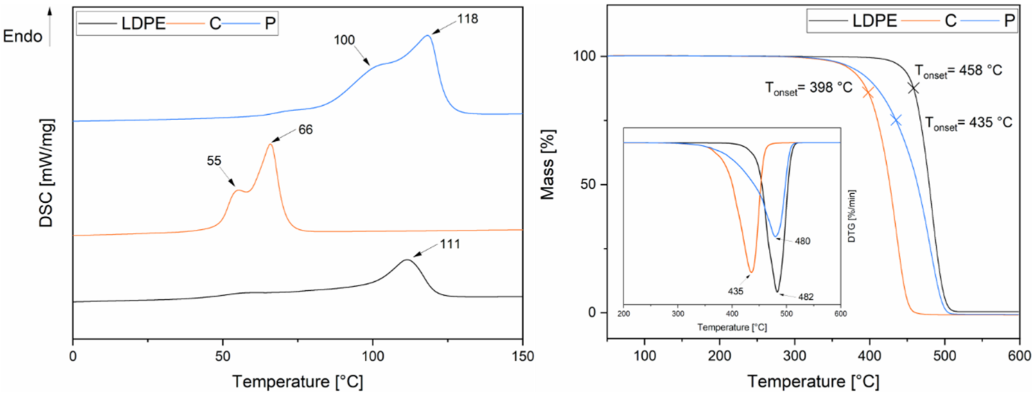


a) b)

**Figure S1.** Thermal properties of the matrix (LDPE) and lubricating additives (C and P) powders: **a**) melting range with peak melting temperatures from the DSC analysis, and **b**) onset decomposition temperatures ($T_{onset}$) from the TG curves and maximum degradation temperatures from the DTG curves.

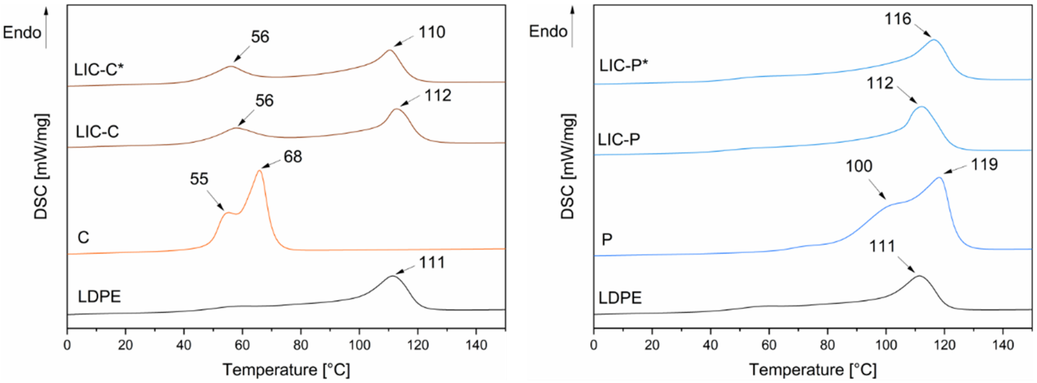


a) b)

**Figure S2.** Curves obtained from the DSC experiments indicating the melting transitions related to **a**) coatings LIC-C and LIC-C* and corresponding feedstock powders (LDPE and C), and **b**) coatings LIC-P and LIC-P* and corresponding feedstock powders (LDPE and P).

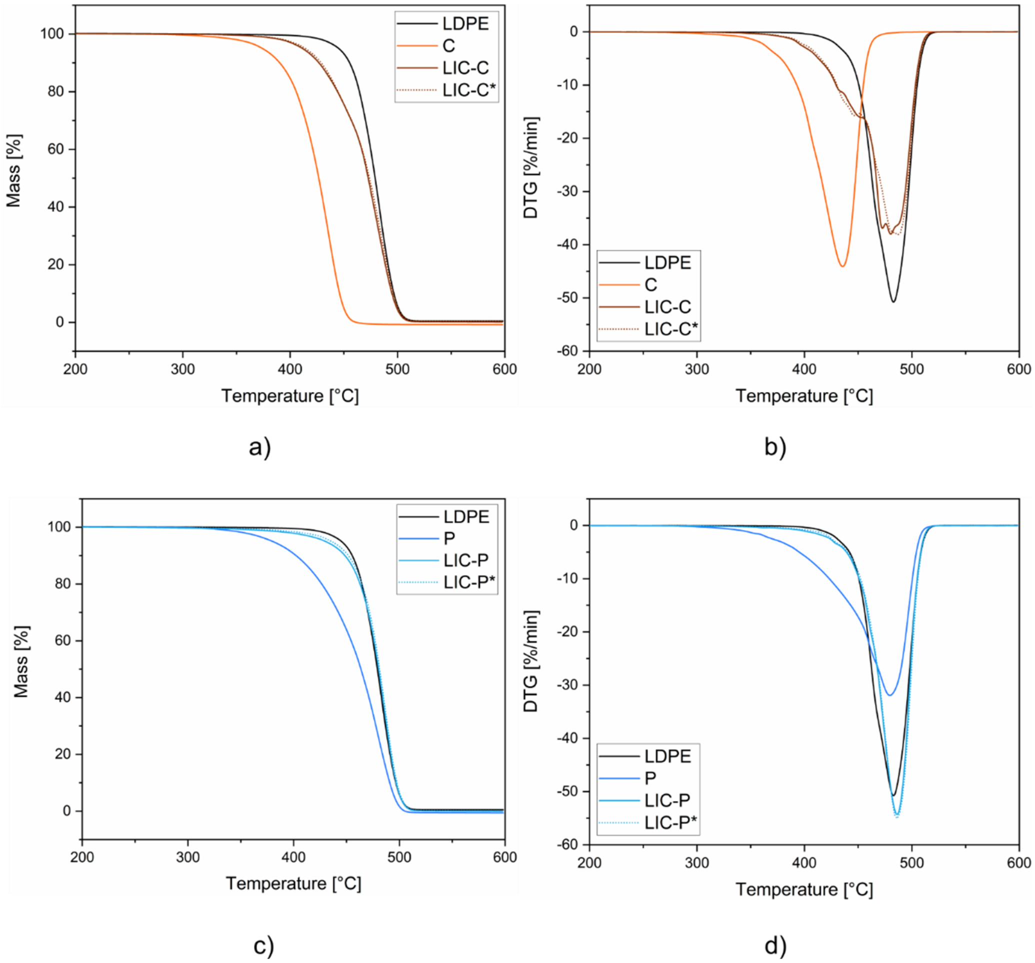


**Figure S3.** Curves obtained from the TG experiments indicating the mass percentage decrease as a function of temperature for **a**) coatings LIC-C and LIC-C* and related feedstock powders (LDPE and C) and **c**) coatings LIC-P and LIC-P* and related feedstock powders (LDPE and P). The degradation peaks evaluated from the first derivative of TG curves (DTG) are related to **b**) LIC-C, LIC-C*, LDPE and C, and **d**) LIC-P, LIC-P*, LDPE and P.

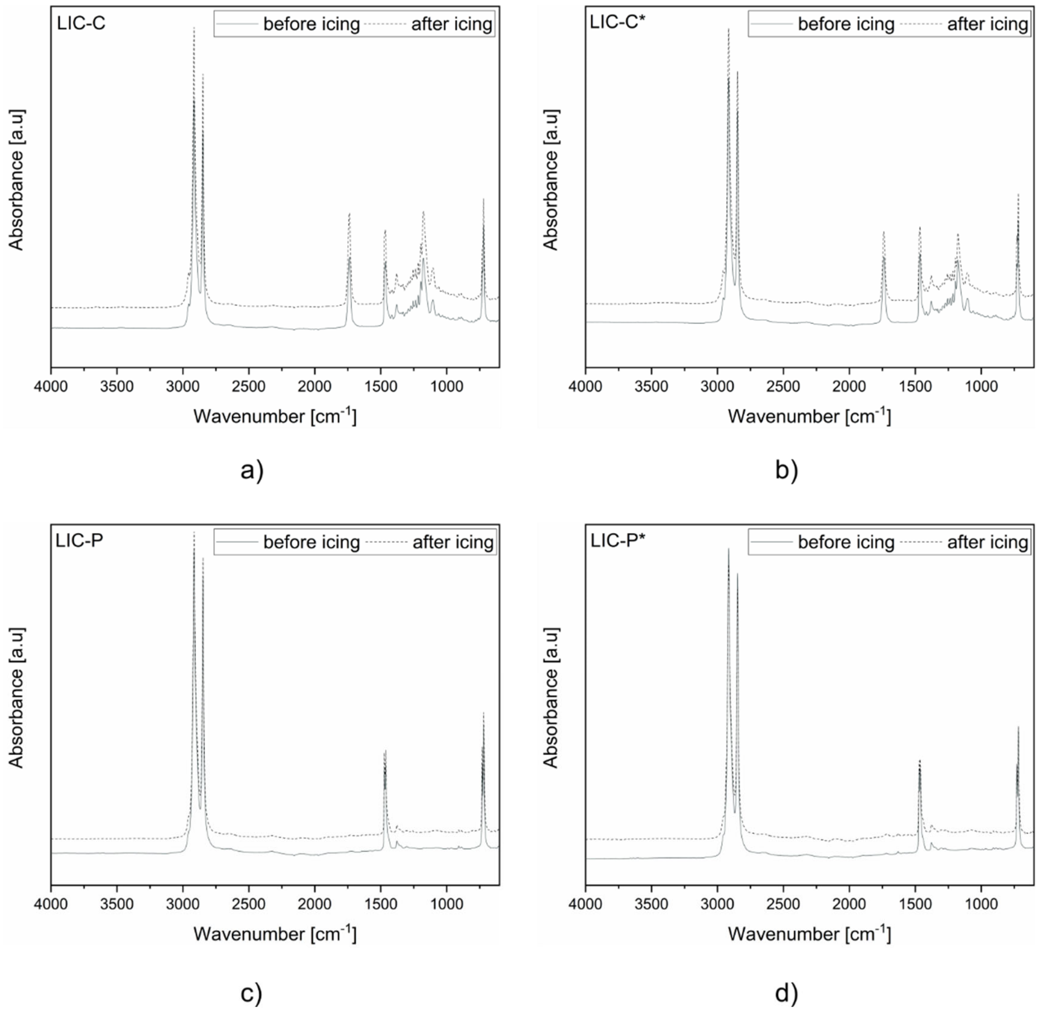


**Figure S4.** The FTIR spectra of the coating surface before icing/deicing cycles (solid line) and after four icing/deicing cycles (dash line). The graphs correspond to samples LIC-C (**a**), LIC-C* (**b**), LIC-P (**c**), and LIC-P* (**d**).

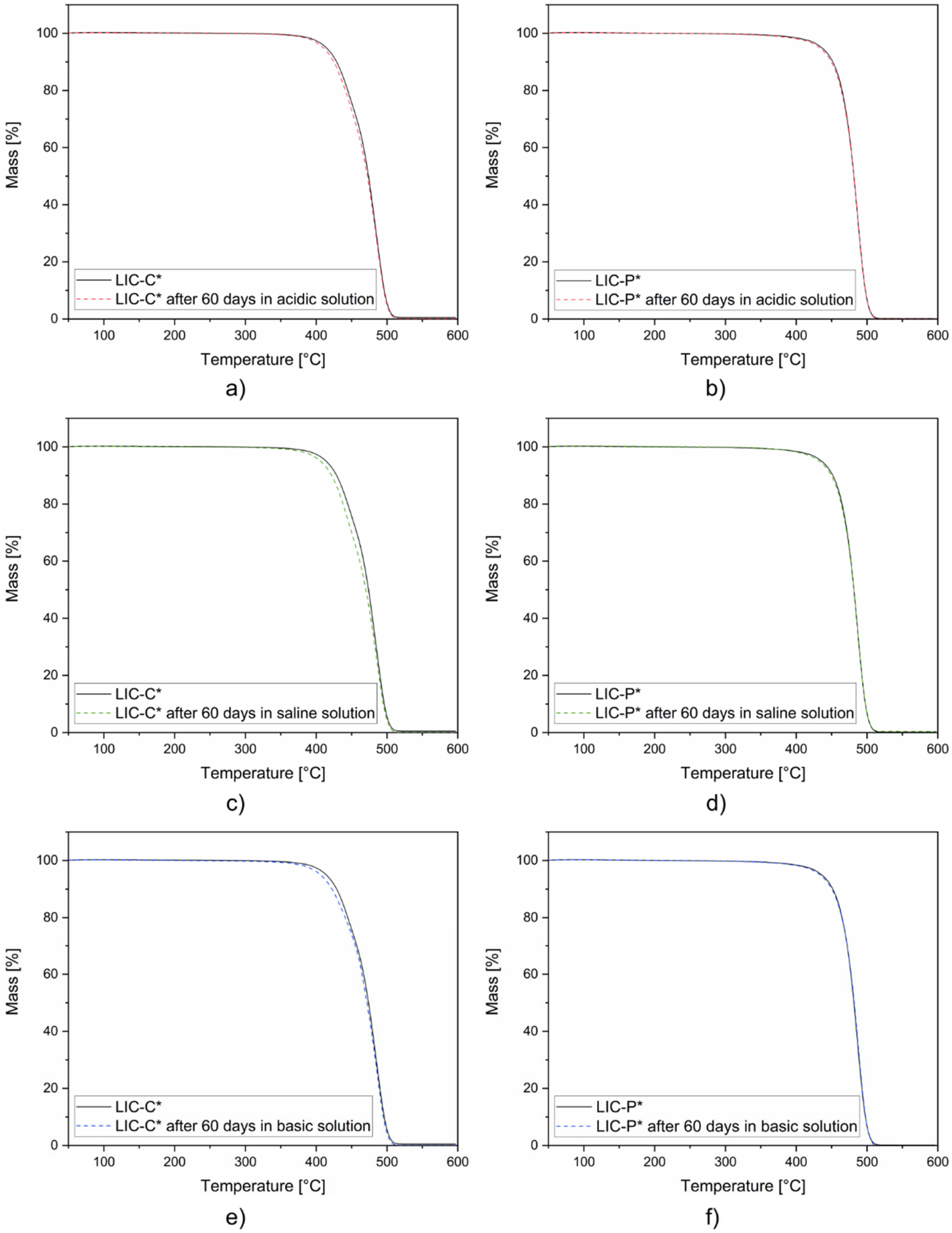


**Figure S5.** Comparison of the thermogravimetric curves of the coatings before and after 60 days of immersion in acidic solution (**a**-**b**), in saline solutions (**c**-**d**), and basic solutions (**e**-**f**).
